# Long-term treadmill exercise attenuates tau pathology in P301S tau transgenic mice

**DOI:** 10.1186/1750-1326-9-54

**Published:** 2014-11-28

**Authors:** Odochi Ohia-Nwoko, Saghi Montazari, Yuen-Sum Lau, Jason L Eriksen

**Affiliations:** Department of Pharmacological and Pharmaceutical Sciences, University of Houston, 521 Science and Research Building 2, 4800 Calhoun Road, Houston, TX 77204 USA; American Association of Colleges of Pharmacy, Alexandria, VA 22314 USA

**Keywords:** Tau pathology, Exercise, Alzheimer’s disease, Neurodegeneration

## Abstract

**Background:**

Recent epidemiological evidence suggests that modifying lifestyle by increasing physical activity could be a non-pharmacological approach to improving symptoms and slowing disease progression in Alzheimer’s disease and other tauopathies. Previous studies have shown that exercise reduces tau hyperphosphorylation, however, it is not known whether exercise reduces the accumulation of soluble or insoluble tau aggregates and neurofibrillary tangles, which are both neuropathological hallmarks of neurodegenerative tauopathy. In this study, 7-month old P301S tau transgenic mice were subjected to 12-weeks of forced treadmill exercise and evaluated for effects on motor function and tau pathology at 10 months of age.

**Results:**

Exercise improved general locomotor and exploratory activity and resulted in significant reductions in full-length and hyperphosphorylated tau in the spinal cord and hippocampus as well as a reduction in sarkosyl-insoluble AT8-tau in the spinal cord. Exercise did not attenuate significant neuron loss in the hippocampus or cortex. Key proteins involved in autophagy—microtubule-associated protein 1A/1B light chain 3 and p62/sequestosome 1 —were also measured to assess whether autophagy is implicated in the exercised-induced reduction of aggregated tau protein. There were no significant effects of forced treadmill exercise on autophagy protein levels in P301S mice.

**Conclusions:**

Our results suggest that forced treadmill exercise differently affects the brain and spinal cord of aged P301S tau mice, with greater benefits observed in the spinal cord versus the brain. Our work adds to the growing body of evidence that exercise is beneficial in tauopathy, however these benefits may be more limited at later stages of disease.

## Introduction

More than 35 million people worldwide are affected by dementia [1], with the leading cause being Alzheimer’s disease (AD), primarily affecting those aged 65 years or older [2]. Central nervous system (CNS) accumulation of hyperphosphorylated tau and amyloid-beta proteins are pathological hallmarks of AD, whereas tau accumulation also occurs in other tauopathies such as frontotemporal dementia (FTD), Pick’s disease (PiD), progressive supranuclear palsy (PSP) and corticobasal degeneration (CBD). These diseases are all characterized by the intraneuronal or glial accumulation of neurofibrillary tangles (NFTs), which are comprised of hyperphosphorylated and aggregated tau protein [3–5]. There are no approved treatments for diseases with only tau inclusions [6], whereas the currently approved drugs for AD temporarily relieve symptoms without altering disease progression [7].

Given the prevalence of these disorders, there is significant scientific and clinical interest in developing new approaches that can be used to prevent disease onset and to attenuate disease progression. Clinical studies suggest that being physically active later in life may be neuroprotective by preserving cognition [8–11], increasing neurotropic factors [12, 13], and maintaining the structural integrity of the brain [14, 15]. Additionally, the neuroprotective benefit of physical exercise has been demonstrated in patients as well as in animal models of AD. Slower declines in the activities of daily living score [16] and measures of functional independence [17], in addition to improvements in cognitive function [18], have been reported in AD patients subjected to aerobic and/or anaerobic forms of exercise. In animal models of AD, exercise has been reported to have a range of beneficial effects, such as a reduction in the build-up of amyloid beta (Aβ) plaques, soluble and fibrillar Aβ peptides [19–21], and improvements in cognitive [19, 22–24] as well as non-cognitive [25] behaviors. In addition to effects on Aβ pathology, there is some evidence of exercise-induced benefits in tauopathy. Steffen and colleagues reported a case of improved motor function and an attenuated rate of brain volume loss in a patient with CBD and PSP after 2.5 and 10 years of exercise [26, 27]. In support of the clinical data, there is also evidence that exercise can alter soluble forms of tau phosphorylation and positively affect behavioral deficits in transgenic mouse models [28, 29].

While much research has been devoted to understanding the mechanisms by which physical activity can reduce or prevent the pathological consequences of toxic Aβ accumulation in AD [19, 22, 23, 30–36], the impact of exercise on the neurodegenerative process in tauopathy is not as well understood. It is known that forced treadmill exercise can decrease tau hyperphosphorylation in *NSE/htau23* mice [28], however this model does not appear to develop neurofibrillary tangles or neurodegeneration [37]. There is also evidence that voluntary wheel running can reduce tau hyperphosphorylation in THY-Tau22 mice [29], but it is not known whether exercise can affect both soluble and sarkosyl-insoluble (or aggregated) forms of tau, which are also pathological hallmarks of tauopathies [4]. Reductions in tau could potentially occur by a number of mechanisms, but there is evidence that autophagy can be induced in the brain by treadmill exercise [38], and recent reports indicate that pharmacological activation of autophagy via trehalose [39] or rapamycin [40] reduces soluble and insoluble tau aggregation in P301S mice. Based on these reports, we hypothesized that the induction of autophagy by exercise could play a role in reducing tau pathology.

In this study, we assessed whether long-term endurance treadmill exercise introduced after the onset of neurodegenerative tauopathy could improve general locomotor function and slow the development of tau neuropathology, possibly through inducing autophagy. Although there is evidence that short-term exercise is beneficial by increasing neurotrophic factors, cell proliferation and synaptic protein levels in the non-diseased rodent brain [41–44], long-term exercise has also been reported to produce similar benefits [45]. However, the effects of either short- or long-term exercise in diseased rodent models are more complex. Previous reports indicate that the benefits of exercise in AD mouse models depend on the duration and type of exercise protocol. For example, short-term, voluntary (wheel-running) exercise did not have a significant impact on Aβ pathology after 3 to 4 weeks [46–48] or 6 weeks [49] of training. Conversely, 3-, 5- and 6-months of voluntary exercise reduced pathology (tau or Aβ) in THY-Tau22 [29], TgCRND8 [19] and 3xTg-AD [50] mice, respectively. Forced treadmill exercise is beneficial after short and long periods of training, as 4 weeks of exercise reduced Aβ pathology in APP/PS1 mice [47], and 3 months of exercise reduced Aβ or tau pathology in *NSE/PS2*[35] and *NSE/htau23*[28, 51] mice. Based on this prior evidence, for the present study we chose to introduce P301S tau transgenic mice—which overexpress the P301S-mutant human tau associated with clinical cases of neurodegenerative tauopathy [52]—to a 12-week forced treadmill exercise regimen that has been reported to benefit a Parkinsonian mouse model of neurodegeneration [53, 54].

## Results

### Exercise increases general locomotor and exploratory behavior in P301S mice

**Figure 1:**
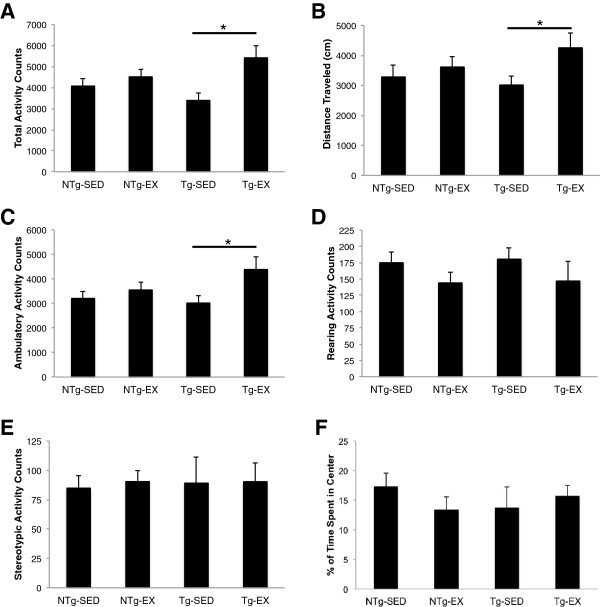
**Open-field locomotor performance enhanced in P301S transgenic mice after 12-weeks of forced treadmill exercise.** Transgenic exercised mice (Tg-EX) showed locomotor improvements in total activity **(A)**, total distance traveled **(B)**, and ambulatory activity **(C)** when compared to transgenic sedentary mice (Tg-SED). No significant changes were observed in rearing **(D)**, stereotypic activity **(E)** and time spent in the center **(F)**. [NTg-SED (n = 10), NTg-EX (n = 8), Tg-SED (n = 6), Tg-EX (n = 8)]. *p < 0.05 = Tg-SED vs Tg-EX. A two-way ANOVA with the Newman-Keuls *post-hoc* was used to detect statistically significant differences.

To assess whether introduction of treadmill exercise could affect animal locomotor activity and exploratory behavior, P301S tau transgenic mice and matching non-transgenic counterparts were tested in the open field. Exercise training resulted in enhanced total exploratory activity in P301S mice. Two-way analysis of variance (ANOVA) revealed a main effect of exercise, [F(1, 32) = 8.1829, p < 0.01]. Newman-Keuls *post-hoc* indicated that the transgenic exercised (Tg-EX) mice had significantly higher total activity versus the transgenic sedentary (Tg-SED) mice (p < 0.01; Figure 1A). We also observed a significant main effect of exercise in total distance traveled, [F(1,32) = 5.6585, p < 0.05], where Tg-EX mice traveled more than Tg-SED mice (p < 0.05; Figure 1B), and ambulatory activity [F(1,32) = 7.9197, p < 0.01], where Tg-EX mice displayed increased ambulation versus Tg-SED mice (p < 0.05; Figure 1C). Exercise-enhanced locomotor behavior in Tg-EX mice was not attributed to either rearing activity, [F(1,32) = 0.2072, p = 0.652] (Figure 1D) or stereotypic activity, [F(1,32) = 0.0573, p = 0.812] (Figure 1E). Additionally, increased locomotor and exploratory activity of Tg-Ex mice was not due to decreased anxiety-like behavior, as all groups spent a similar amount of time in the center of the testing chamber, [F(1,32) = 0.1514, p = 0.700] (Figure 1F). There were no significant differences in locomotor or exploratory performance between the non-transgenic exercised (NTg-EX) and non-transgenic sedentary (NTg-SED) groups in total activity (p = 0.446; Figure 1A), total distance traveled (p = 0.542; Figure 1B), and ambulation (p = 0.494; Figure 1C).

### Exercise training reduces total and hyperphosphorylated tau immunofluorescence in P301S mice

**Figure 2:**
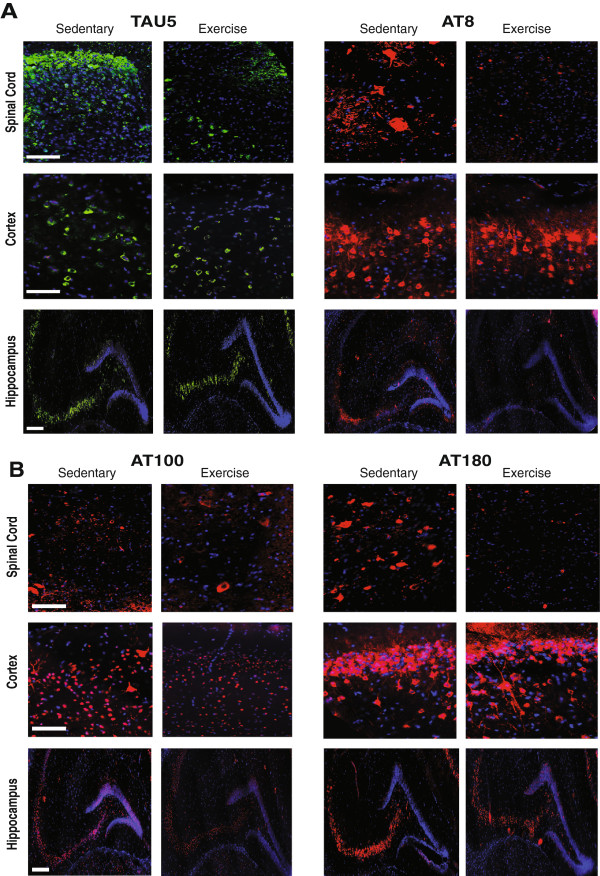
**Total and phosphorylated tau immunofluorescence is reduced in the spinal cord and brain of P301S transgenic mice after 12-weeks of forced treadmill exercise.** Representative images of total (TAU5, **A**) and phosphorylated tau (AT8, **A**; AT100 and AT180, **B**) immunofluorescence in the spinal cord (dorsal horn), cortex (layers I-II), and hippocampus. For spinal cord and cortex images, scale bars represent 100 μm. For hippocampus images, scale bar represents 200 μm. Green = TAU5; red = AT8, AT100, or AT180; blue = DAPI.

**Figure 3:**
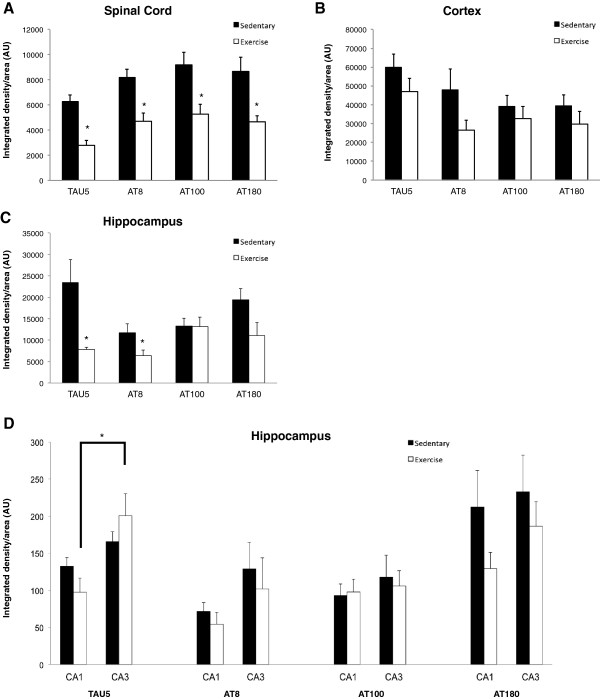
**Total and phosphorylated tau immunofluorescence is reduced in the spinal cord and brain of P301S transgenic mice after 12-weeks of forced treadmill exercise. A)** Total (TAU5) and phosphorylated (AT8, AT100, and AT180) tau were significantly reduced in the spinal cord of the exercised P301S mice. **B)** No statistically significant changes in total and phosphorylated tau immunofluorescence were observed in in the cortex exercised versus sedentary transgenic mice. **C)** Significant reductions in TAU5 and AT8 were observed in the hippocampus of exercised P301S mice. **D)** Exercise significantly affected TAU5 immunofluorescence in the CA1 versus the CA3 of the hippocampus. [n = 6-8 per group]; (*) denotes a significant difference (p < 0.05). A Student’s t-test was used to detect statistically significant differences.

We used a Student’s t-test to compare Tg-SED and Tg-EX groups and observed a significant reduction in total (TAU5) and phospho-tau immunofluorescence in the spinal cord (p < 0.05; Figures 2 and 3A) and hippocampus (p < 0.05; Figures 2 and 3C) of Tg-EX mice (Figure 3A,C) after exercise. Tau phosphorylated at sites Ser202 and Thr205 (AT8) was also significantly reduced in the spinal cord (p < 0.05; Figure 3A) and hippocampus (p < 0.05; Figure 3C), in the Tg-EX mice. Additionally, a significant reduction in tau phosphorylated at sites Thr212/Ser214 (AT100) and Thr231 (AT180) was observed in the spinal cord (p < 0.05; Figure 3A), while a reduction in AT180 in the hippocampus almost reached statistical significance (p = 0.058; Figure 3C). No significant changes in total and phosphorylated tau were observed in the cortex (Figures 2 and 3B). When the CA1 and CA3 subregions were separately measured, exercise appeared to significantly reduce TAU5 immunofluorescence in the CA1 versus the CA3 of the hippocampus. We also observed marginal reductions in AT180 in the both sub regions after exercise (Figure 3D).

### Exercise has differential effects on soluble and insoluble tau in the spinal cord and brain of P301S mice

**Figure 4:**
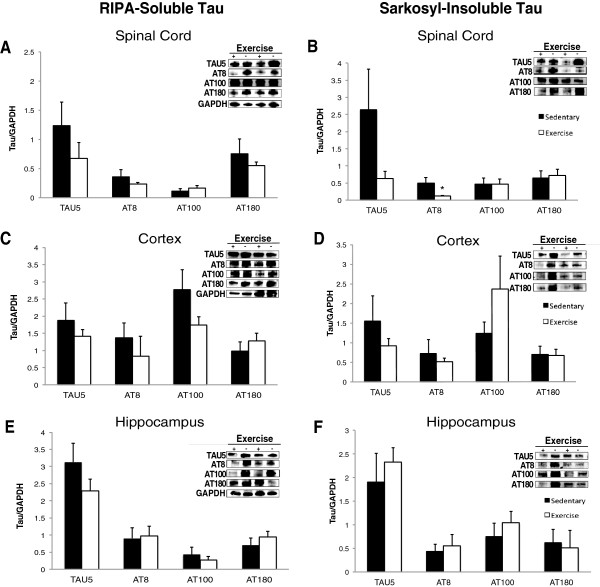
**RIPA-soluble and sarkosyl-insoluble tau levels in the spinal cord, cortex, and hippocampus of P301S transgenic mice.** RIPA-soluble total (TAU5) and phosphorylated (AT8, AT100, AT180) protein quantification via Western blot analysis of the **A)** spinal cord, **C)** cortex, and **E)** hippocampus revealed no significant reductions in exercised versus sedentary P301S mice. Sarkysol-insoluble total and phosphorylated Western blot analysis of the **D)** cortex and **F)** hippocampus did not reveal significant reductions in exercised versus sedentary P301S mice, however the **B)** spinal cord revealed a significant reduction in AT8-tau. [n = 6-8 per group] (*) denotes a significant difference (p < 0.05). Student’s t-test was used to detect statistically significant differences.

**Figure 5:**
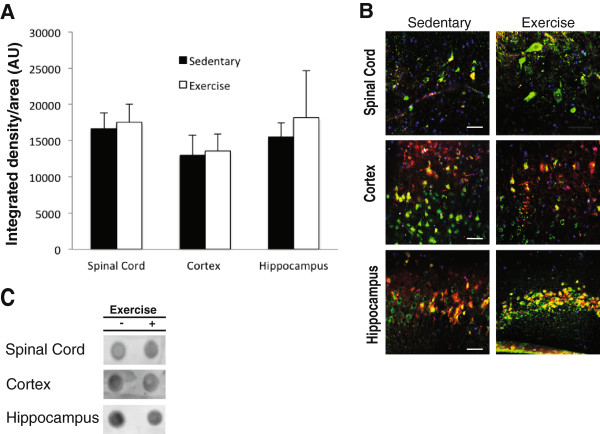
**Soluble tau oligomers are present in the spinal cord and brain of P301S mice. A)** Dot blot analysis indicates similar levels of oligomeric tau in the spinal cord, cortex, and hippocampus. **B)** Representative images of immunofluorescence of the spinal cord, cortex and hippocampus revealed similar T22 staining in sedentary versus exercised mice. Scale bar represents 50 μM for all images. **C)** Representative dot blots of T22 in the spinal cord, cortex, and hippocampus. [N = 4 per group]. Blue, DAPI; green, TAU5; red, T22. A Student’s t-test was used to detect statistically significant differences.

In order to assess whether exercise could affect the levels of pathological tau, we performed sequential RIPA and sarkosyl-extractions to quantify levels of soluble and insoluble tau protein, respectively [55]. A Student’s t-test was used to analyze differences between Tg-SED and Tg-EX groups. We observed marginal reductions in total soluble tau protein in the spinal cord (45% decrease), cortex (25% decrease), and hippocampus (27% decrease) in Tg-EX versus transgenic sedentary (Tg-SED) mice. (TAU5: Figure 4A, C and E). Total insoluble-tau protein was also reduced in the spinal cord (76% decrease) and cortex (41% decrease) but not in the hippocampus (TAU5: Figure 4B, D and F) of Tg-EX versus Tg-SED mice. Soluble AT8-tau was reduced in Tg-EX versus Tg-SED mice in the spinal cord (35% decrease) and cortex (40% decrease), but not the hippocampus (Figure 4A, B and C); however, a significant reduction in insoluble AT8-tau was observed in the spinal cord (p < 0.05; Figure 4B). No significant reductions in insoluble AT8-tau were observed in the cortex or hippocampus after exercise (Figure 4D,F). Exercise did not appear to significantly influence the levels of soluble and insoluble AT100-tau in the spinal cord of Tg-EX compared to Tg-SED mice (Figure 4A and B). In the cortex, we observed a 37% reduction in soluble AT100-tau and an unexpected 48% increase in insoluble AT100-tau in the cortex of Tg-EX mice (Figure 4C,D). No significant reductions in soluble or insoluble AT100-tau were observed in the hippocampus (Figure 4E,F). We also did not observe any reductions in soluble or insoluble AT180-tau in the spinal cord, cortex and hippocampus of Tg-EX versus Tg-SED mice (Figure 4A-F). Dot blot analysis revealed that exercise did not affect the levels of tau oligomers in any region; mice from the Tg-EX group displayed similar levels of oligomeric tau in the spinal cord, cortex and hippocampus (Figure 5A,C).

### Exercise does not impact neuronal cell number in the spinal cord or brain of P301S mice

**Figure 6:**
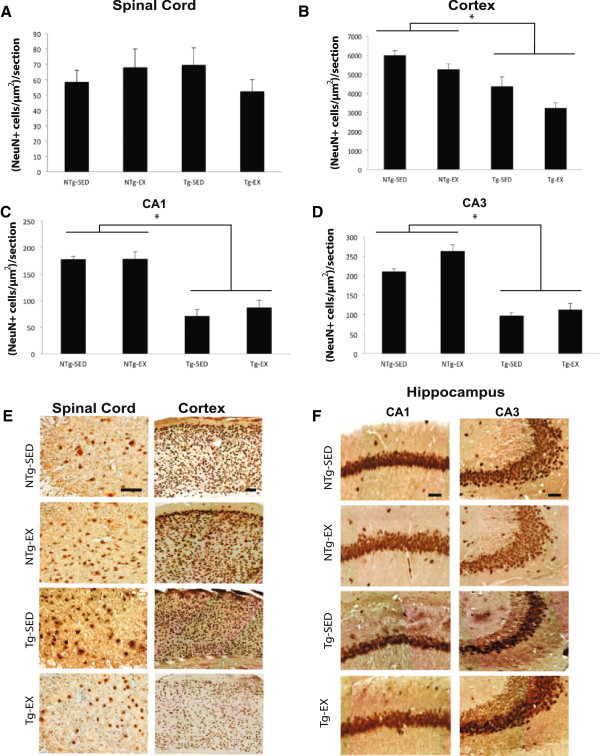
**Forced treadmill exercise does not affect neuronal cell counts in the spinal cord and brain. A)** Spinal cord motor neuron counts were not significantly different across all groups. **B)** Transgenic mice displayed significant neurodegeneration in the cortex compared to non-transgenic controls. **C)** Transgenic mice displayed significant neurodegeneration in the CA3 of the hippocampus compared to non-transgenic controls. **D)** Transgenic mice displayed significant neurodegeneration in the CA1 of the hippocampus compared to non-transgenic controls. **E)** Representative images of the spinal cord and cortex. Scale bars represent 50 μm for spinal cord and 100 μm for the cortex. **F)** Representative images of the CA1 and CA3 subregions of the hippocampus. Scale bar represents 25 μm. [NTg-SED (n = 6), NTg-EX (n = 8), Tg-SED (n = 6), Tg-EX (n = 8)]; (*) denotes a significant difference (p < 0.05). A two-way ANOVA with the Tukey’s HSD *post-hoc* was used to detect statistically significant differences.

Quantification of motor neurons in the spinal cord revealed no significant difference in cell number between all 4 groups, with two-way ANOVA indicating no main effect of genotype, [F(1,28) = 0.2212, p = 0.642] or exercise, [F(1,28) = 0.8274, p = 0.371] (Figure 6A). Cortical neurodegeneration was observed in P301S mice, with a main effect of genotype, [F(1, 27) = 27.8664, p < 0.01]. Tukey’s HSD *post hoc* revealed that the Tg-SED group had less NeuN-positive cells than the NTg-SED group (p < 0.05; Figure 6B). Similarly, the Tg-EX group had less NeuN-positive cells than the NTg-EX group (p < 0.01; Figure 6B). P301S transgenic mice displayed neurodegeneration in the cornus ammonis (CA) 3 of the hippocampus, with a main effect of genotype, [F(1, 24) = 41.5653, p < 0.01]. Tukey’s HSD *post hoc* revealed that Tg-SED mice had significantly fewer NeuN-positive cells than NTg-SED mice (p < 0.05; Figure 6D) and the Tg-EX mice had fewer NeuN-positive cells than the NTg-EX mice (p < 0.01; Figure 6D). Neurodegeneration was also observed in the CA1 of the hippocampus of P301S mice [F(1, 27) = 57.0844, p < 0.01] with both Tg-SED and Tg-EX mice displaying significantly fewer NeuN-positive cells than their NTg counter parts (p < 0.01; Figure 6C).

### Exercise and autophagy-related proteins in P301S mice

**Figure 7:**
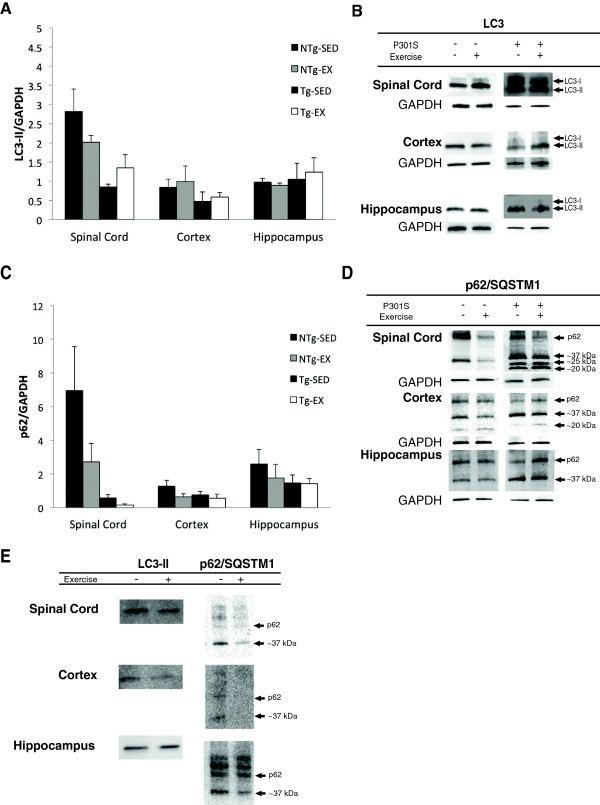
**LC3-II and p62/SQSTM1 Western blot analysis. A)** There were no significant effects of exercise on LC3-II levels in P301S or non-transgenic mice in the spinal cord, cortex, and hippocampus. [N = 3-4 per group] **B)** Representative western blots of LC3-II. **C)** There were no significant effects of exercise on p62/SQSTM1 levels in P301S or non-transgenic mice in the spinal cord, cortex, and hippocampus. Intense 37, 25, and 20 kDa bands were observed in P301S mice, an indication of proteolytic cleavage. [N = 3-4 per group] **D)** Representative western blots of p62/SQSTM1. **E)** Representative western blots of sarkosyl-insoluble LC3-II and p62/SQSTM1 in the hippocampus, cortex, and spinal cord.

We did not observe any significant effects of exercise on protein levels of microtubule-associated protein 1A/1B light chain 3-II (LC3-II) and p62/sequestosome 1 (p62/SQSTM1) of the NTg versus Tg groups. A Student’s t-test was used to compare differences between groups. In the spinal cord, protein levels of LC3-II (p = 0.2380) and p62 (p = 0.1839) were not significantly different in NTg-SED versus NTg-EX mice (Figure 7). Similarly, no differences were observed in the cortex (LC3-II: p = 0.7610; p62: p = 0.1607) and hippocampus (LC3-II: p = 0.4773; p62: p = 0.6878) of NTg-SED versus NTg-EX mice (Figure 7). We also did not observe any significant differences in the spinal cord (LC3-II: p = 0.2040; p62: p = 0.16901), cortex (LC3-II: p = 0.7037; p62: p = 0.5223) and hippocampus (LC3-II: p = 0.7632; p62: p = 0.9497) of Tg-SED versus Tg-EX mice (Figure 7). While not statistically significant, lower levels of both microtubule-associated protein 1A/1B light chain 3-II (LC3-II) and p62/sequestosome 1 (p62/SQSTM1) were observed after exercise in non-transgenic mice. We did not observe strong LC3-I bands in the cortex and hippocampus (Figure 7A), which could be due to the greater sensitivity of the anti-LC3 antibody to LC3-II over LC3-I in some cases [56]. When probing for p62/SQSTM1 in the spinal cord, we observed lower levels in Tg-EX mice versus Tg-SED mice and the presence of a stronger 37 kDa band in the spinal cord, cortex, and hippocampus of transgenic versus non-transgenic mice (Figure 7D). The 37 kDa band is known to be a product of caspase cleavage *in vitro*[57, 58]. Additionally, we also observed the presence of 25 and 20 kDa bands in the spinal cords of transgenic mice, whereas these bands were not present in the non-transgenic mice (Figure 7D). Additionally, we observed accumulation of LC3-II and p62/SQSTM1 in the sarkosyl-insoluble fraction of the spinal cord, cortex and hippocampus of P301S mice (Figure 7E).

## Discussion

Pathological tau and/or Aβ accumulation occurs in Alzheimer’s disease and other tauopathies. There are numerous reports that have focused on the exercise-induced reductions of Aβ pathology and behavioral impairments in animal models of Alzheimer’s disease [19–25, 32, 34]. It has been reported that exercise can also reduce soluble hyperphosphorylated tau in mouse models of tauopathy [28, 29]. In this report, we investigated whether long-term (12 weeks) forced treadmill exercise could attenuate or prevent the progression of tauopathy in mice that overexpress human P301S-mutated tau. The P301S mutation is associated with familial forms of FTD [3, 59, 60], and is known to cause hyperphosphorylation, aggregation and filament formation of tau [61], which decreases the affinity of tau for microtubules and leads to subsequent neurodegeneration and the development of NFTs. P301S tau transgenic mice develop extensive tau pathology in the spinal cord at 6 months of age [39, 62] accompanied by neurogenic muscle atrophy [52], which results in a progressive decline in locomotor function. While our P301S mice had slightly lower activity in the open field, we did not observe a profound deficit in general locomotor and exploratory activity in our 10-month old P301S mice compared to the non-transgenic mice, potentially due to phenotypic drift that has been previously reported in this mouse line [63–65].

We observed significant tau pathology in our 10-month old mice and forced exercise training significantly enhanced locomotor and exploratory activity in Tg-EX versus Tg-SED mice. Our observations are in accordance with recent clinical evidence [26, 27] showing that a patient diagnosed with CBD and PSP that participated in a regular exercise program (including treadmill training) for 10 years displayed reduced fall frequency, as well as improved balance and ambulation after exercise training [27]. The enhancement of locomotor ability we observed could be attributable to a variety of factors. We observed significant reductions in tau hyperphosphorylation and aggregation in the spinal cord, an indication that the progression of tau pathology was attenuated by exercise. Hyperphosphorylation and aggregation of tau are associated with synapse loss [52] and altered synaptic function [66] in P301S mice, so it is possible that treadmill exercise prevented these alterations in the spinal cord, restoring adequate neurotransmission at the neuromuscular junction. Treadmill exercise elevates the expression of synaptophysin and synapsin 1 [67–73], as well as post-synaptic density protein-95 [68, 74, 75], supporting the notion that exercise enhances synaptic neurotransmission. A reduction in tau pathology via exercise could result in an increase in expression of synaptic proteins, or this increase could occur directly via forced exercise, resulting in enhanced synaptic transmission in the spinal cord and improved locomotor function.

Pre-neurofibrillary tangles are comprised in part, by pThr231 tau, while extracellular and intracellular neurofibrillary tangles (comprised of mostly filamentous tau) are stained with antibodies that recognize phosphorylation at sites Ser202/Thr205 (AT8) and Thr212/Ser214 (AT100) in Alzheimer’s disease [76]. In our study, we observed reduced total and hyperphosphorylated tau in the lumbar spinal cord and hippocampus, showing a significant reduction of phosphorylated tau Ser202/Thr205 (AT8) in the spinal cord and hippocampus, and Thr231 (AT180) and Thr212/Ser214 (AT100) in the spinal cords of Tg-EX mice. These findings indicate that forced treadmill exercise attenuates the progression of neurofibrillary tangle formation in the spinal cord and reduces filamentous tau in the hippocampus of P301S mice. Our data are consistent with the results published by Leem and colleagues [28], who reported a reduction in AT8-positive immunoreactivity in the hippocampus after forced treadmill exercise in Tg-NSE/*htau23* mice. However, our results are in contrast to that of previous studies using other mouse models of tauopathy, where a reduction in AT100 [29] and not AT8-positive immunoreactivity [29, 77, 78] was observed in the hippocampus after voluntary wheel running exercise. The differences in experimental outcomes could be attributed to different choices of exercise modality [79], where forced treadmill exercise maybe more beneficial in tauopathy versus voluntary exercise. Additionally, different choice of experimental mouse model used in the present study versus the aforementioned reports could also be a source of the discrepancy in results.

Despite evidence that exercise can reduce tau hyperphosphorylation in mice [28, 29], it is not known whether forced treadmill exercise can reduce insoluble tau accumulation, which is a pathological characteristic of tauopathies [4]. To address this question, we analyzed RIPA-soluble and sarkosyl-insoluble forms of tau protein in the spinal cord and brain of P301S mice. Sarkosyl protein extractions are routinely used to isolate aggregated paired helical filaments of tau, which are the primary constituents neurofibrillary tangles [80]. While we observed marginal reductions in total tau for RIPA-soluble and sarkosyl-insoluble tau pools in the spinal cord, there was a significant reduction in sarkysol-insoluble AT8-tau, suggesting that forced treadmill exercise reduces filamentous tau accumulation. We observed only marginal reductions in soluble or insoluble tau protein in the hippocampus and cortex. These observations are similar to our histological data, where no significant changes in total or hyperphosphorylated tau were observed in the cortex as well as in AT100 and AT180 in the hippocampus. In addition to filamentous tau, we also analyzed the levels of soluble tau oligomers, which are thought to be toxic participants in neurodegenerative tauopathies [81, 82]. We found that P301S tau mice express soluble oligomeric tau; however, exercise did not appear to significantly affect oligomeric tau levels in the brain and spinal cord.

Our results suggest that forced treadmill exercise reduces total and phosphorylated insoluble tau in the spinal cord, but only moderate changes occur in the brain. In accordance with these results, we observed a significant degree of cell loss in the hippocampus and cortex that was not alleviated by our exercise regimen. In the hippocampus, the CA1 and CA3 regions of P301S mice displayed neurodegeneration, which is consistent with previous reports in this mouse model [52, 77, 83]. Both hippocampal regions have extensive connections with the entorhinal cortex (EC) [84, 85], where significant tau pathology and cell loss is also observed [52]. Significant neurodegeneration in the CA regions could result from synaptic propagation of tau pathology from the EC [86, 87] and/or degeneration of EC afferents [88, 89]. Since the dentate gyrus (DG) also relies on its connections with the EC [84, 85], it is plausible that tau propagation from the DG to the CA3 (and the CA1 via the Schaffer collaterals), or deafferentation could also impact the neurodegenerative process in CA hippocampal regions.

Given that we did not see significant reductions in hippocampal and cortical soluble or insoluble tau in Tg-EX mice, neurodegenerative tau pathology in the hippocampus and cortex may have progressed to a stage that could not be mitigated with 12 weeks of forced treadmill exercise. Previous reports have suggested that forced treadmill exercise may not reverse or prevent some diseases [90, 91] and in some cases can exacerbate disease progression [92, 93] possibly because of increased stress associated with the treadmill protocol. However, our exercise regimen did not appear to worsen tau pathology in the brains of P301S mice.

Neurodegenerative diseases are characterized by the accumulation of aggregated proteins, an indication that there is either increased production or inefficient elimination of dysfunctional or misfolded proteins that results in perturbed proteostasis. Both autophagy and the ubiquitin-proteosome system have been implicated in abnormal protein accumulation associated with neurodegenerative processes [94, 95]. Therefore, our final aim was to investigate whether autophagy is a possible mechanism by which tau pathology was mitigated by forced treadmill exercise. Autophagy activation is characterized, in part, by increased production of LC3-II and increased degradation of p62/SQSTM1[38, 96] relative to baseline levels. We observed that P301S and non-transgenic mice introduced to forced treadmill exercise did not have significantly increased levels of LC3-II in the spinal cord and brain when compared to their sedentary counterparts. The levels of p62/SQSTM1 were marginally reduced after exercise in the spinal cords of both non-transgenic and P301S mice, while no changes were observed in the hippocampus and cortex; therefore, the reduced levels of p62/SQSTM1 we observed in the spinal cord of NTg-EX and Tg-EX mice could be an indication of autophagy induction. We observed significant reductions of insoluble AT8-tau in the spinal cord, which could be a result of degradation of tau aggregates by exercise-induced autophagy. In support of this notion, pharmacological activation of autophagy via rapamycin [40] and trehalose [39, 97] reduces insoluble AT8-tau in P301S mice. In addition to aggregated tau, autophagy is also known to promote the degradation of several aggregated proteins associated with neurodegenerative disease, including Aβ [98], huntington [99, 100], and alpha-synuclein [100, 101]. However, we did not observe reductions in insoluble phosphorylated tau or p62/SQSTM1 in the brain.

In this study, P301S mice displayed multiple lower molecular weight p62/SQSTM1 bands (37, 25 and 20 kDa) in the spinal cord. This observation is consistent with increased caspase or calpain cleavage [57, 58], which suggests that the P301S mutation could result in a selective disruption of the autophagic processes, potentially attributable to the loss of polyubiquitin- and LC3-binding regions of p62/SQSTM1 following proteolytic cleavage [57]. The loss of the polyubiquitin-binding region of p62/SQSTM1 may also disrupt proteasomal degradation of tau, as p62/SQSTM1 participates in the shuttling of ubiquitinated tau to the proteasome [102]. It has been hypothesized that impaired proteasomal degradation of soluble tau could lead to its toxic accumulation [103, 104], thus impairment in the ubiquitin-proteasome system in P301S tau mice is also conceivable. Therefore, our observations are consistent with previous observations that disruptions in protein degradation systems may occur in neurodegenerative tauopathy [105], which is in line with previous reports of autophagy disruption in other neurodegenerative diseases [106–109].

Since exercise is known to produce a variety of positive changes in the CNS, we cannot exclude the possibility that other mechanisms may underlie the reductions in tau pathology we observed. Several types of exercise are known to increase the level of neurotrophins in the CNS, particularly, brain-derived neurotrophic factor (BDNF) [110–114], which increases neuronal survival and differentiation [115]. Elevated levels of BDNF also decrease tau phosphorylation via the PI3K-Akt pathway by decreasing the activity of glycogen synthase kinase-3β (GSK-3β), a major tau kinase [116, 117]. In the present study it is possible that exercise-induced increase in BDNF levels (and decreased GSK-3β activity) resulted in the attenuation tau phosphorylation we observed. Indeed, recent evidence suggests that forced treadmill exercise elevates the levels of inactivated phospho-GSK-3β in the brain [74, 118]. This evidence supports an alternate mechanism by which exercise can modulate the progression of tau pathology, which warrants further investigation in future studies with P301S mice.

In conclusion, our work demonstrates that 12 weeks of forced treadmill exercise significantly attenuates tau pathology in the spinal cord and has moderate effects in the brains of older P301S tau mice, but that treadmill exercise does not prevent the progressive underlying neurodegeneration associated with tauopathy when introduced at later ages. Given that our results show exercise modulates tauopathy, our observations suggest that there may be a critical window whereby exercise can have the greatest impact on disease progression; the introduction of forced treadmill exercise before the development of severe pathology may be more beneficial. Our results also support the possibility for a role of autophagy in the exercise-induced reduction of tauopathy, and that autophagy and/or proteasomal dysfunction in P301S tau mice may contribute to the development of tau pathology.

## Materials and methods

### Animals

The P301S tau transgenic mouse expresses human tau (1N4R) with a P301S mutation [52]. These mice display progressive neurofibrillary tangle (NFT) pathology and neurodegeneration in the brain and spinal cord, developing lower-limb weakness around 6 months of age. P301S tau mice (7–8 months old), and their age-matched non-transgenic controls were individually housed in the animal facility at the University of Houston. Mice were housed in a climate-controlled room (25°C) on a 12/12 h light/dark cycle and given food and water *ad libitum*. All experiments were approved by the University of Houston Institutional Animal Care and Use Committee and implemented following the National Research Council’s Guide of The Care and Use of Laboratory Animals.

### Forced treadmill exercise

Mice were designated to four groups: exercised (Tg-EX) and sedentary (Tg-SED) P301S mice along with their non-transgenic exercised (NTg-EX) and sedentary (NTg-SED) counterparts. Beginning at 7 months of age, mice ran on a six-lane motorized treadmill (Columbus Instruments, Columbus, OH, USA) 5 days/week for 40 min/day for 12 weeks. The mice in the exercise groups were trained on treadmill running with a speed up to 15 m/min (5 min at 6 m/min, 5 min at 9 m/min, 20 min at 12 m/min, 5 min at 15 m/min, and 5 min at 12 m/min) with a 0° inclination. All exercised mice ran 450 m per day with a total of 27 km for the 12 week duration of the study. This exercise protocol has been shown to produce cardiorespiratory and metabolic adaptations similar to those seen in humans while exercising [53, 54, 119]. The sedentary mice were brought to the exercise facility in order to expose these mice to the same conditions as the exercised mice. In order to ensure that all mice were able to complete the exercise protocol, mice were qualitatively evaluated throughout the duration of exercise trials.

### Open field activity

Mice were tested 48 h after completion of 12 weeks of endurance treadmill exercise. Mice were placed in the center of a 60 × 40 cm Plexiglas box and allowed to explore the area for 30 min. Open field activity was measured in standard lighting conditions using a computer-operated Opto-Varimex Micro Activity Meter v2.00 system, as previously described [120]. Briefly, each Plexiglas testing chamber contained sensors with 8-infared light emitting diodes and 8 phototransistors that emit and detect infared light beams. Movement was detected by beam breaks, and the Opto-Varimex program recorded total activity counts, distance traveled, ambulatory activity counts, rearing activity counts and stereotypic activity counts.

### Tissue extraction

Mice were sacrificed under carbon dioxide (CO_2_) anesthesia and the brains and spinal cords were dissected. Each hemibrain and spinal cord was placed in Accustain (a proprietary formalin-free fixative from Sigma-Aldrich, St. Louis, MO, USA) for tissue fixation for 24 hours at 4°C. After Accustain fixation, the hemibrain and spinal cord were stored in 70% ethanol at 4°C for paraffin processing. The other half of each brain and the cervical spinal cord were snap frozen stored at -80°C for biochemical processing. Tissues were homogenized in cold RIPA lysis and extraction buffer (Thermo-Fisher Scientific, Rockford, IL, USA) containing protease and phosphatase inhibitors. Samples were then centrifuged at 20,000 × *g* for 20 min at 4°C. The pellet was discarded, and a portion of the RIPA lysate was used for biochemical analysis. Sarkosyl-insoluble tau was isolated following previous reports [55], with RIPA supernatants adjusted to 1% sarkosyl. Samples were incubated for 1 hr at room temperature and then spun at 100,000 × *g* at room temperature. The supernatants were discarded and the pellets were resuspended in O+ buffer (62.5 mM Tris–HCl, 10% glycerol, 5% 2-mercaptoethanol, 0.1% SDS, phosphatase and protease inhibitors). Samples were then boiled for 3 min and kept at -20°C for Western blot analysis.

### Immunofluorescence and image analysis

Brain and lumbar spinal cord tissue was paraffin processed and sectioned at a 10 μm thickness. Immunofluorescence was performed on the lumbar spinal cord and hemibrain sections using the TAU5 antibody (anti-TAU, 1:500, Invitrogen) that recognizes phosphorylated and non-phosphorylated tau and phospho-dependent antibodies: AT8 (anti-pSer202/Thr205, 1:500, Thermo Scientific), AT100 (anti-pSer212/Thr214, 1:500, Thermo Scientific), and AT180 (anti-pThr231, 1:500, Thermo Scientific). Additionally, double immunofluorescence with a subset of P301S mice (n = 4 mice per group) with TAU5 (1:500) and the T22 antibody (1:200, kindly donated by Dr. R. Kayed), which specifically recognizes oligomeric forms of tau [121]. For image analysis, up to six equidistant sections were chosen per animal, corresponding to plates 42 to 49 in the brain [122] and L1 to L6 of the spinal cord [123]. Integrated density analysis was measured by a blinded observer with NIH Image J software to quantify total immunofluorescence staining per relevant area (spinal cord, cortex, or hippocampus) for each section, using a method by Barbero-Camps and colleagues to quantify immunofluorescence [124].

### Immunohistochemistry and image analysis

Brain and lumbar spinal cord tissue was paraffin processed and sectioned at a 10 μm thickness and the same brain plate and spinal cord regions used in the immunofluorescence analysis were used. Immunohistochemistry was performed on equidistant sections from the lumbar spinal cord (n ≥ 4 sections per animal) and hemibrain (n ≥ 4 sections per animal) using anti-NeuN (1:1000, Millipore). Sections were incubated in primary antibody overnight at 4°C, incubated with horseradish peroxidase-labeled secondary antibody and stained with DAB. The number of positively-stained neurons with a clearly identifiable nucleus were quantified by a blinded observer using NIH Image J software to determine the number of alpha motor neurons in the ventral horn of the spinal cord as well as neurons in the cortex. For the hippocampus, a 200 μm × 1000 μm rectangular region of the CA3 and CA1 was quantified manually [52].

### Western blot and dot blot analysis

Spinal cord, hippocampus, and cortex samples from both RIPA and sarkosyl extractions were resolved by SDS-PAGE or dot blot. Blots were probed with tau antibodies [TAU5, (1:1000), AT8 (1:1000), AT100 (1:1000) and AT180 (1:250)], and autophagy-related antibodies (anti-LC3, 1:500, Novus Biologicals; anti-p62, 1:1000, BD Biosciences). After overnight incubation at 4°C, blots were incubated in horseradish peroxidase-labeled secondary antibodies and visualized with an ECL substrate kit (Amersham). Band densities were analyzed with NIH Image J software and band values were normalized to glyceraldehyde-3-phosphate dehydrogenase (GAPDH). Dot blots were prepared by pipetting 1.2 ul of each sample in each square of a nitrocellulose membrane and allowed to dry for 30 minutes. Blots were incubated with T22 (1:250), overnight at 4°C followed by incubation with a horseradish peroxidase-labeled secondary and visualization with ECL. Dot intensities were analyzed with NIH Image J software.

### Statistical analysis

A Student’s t-test was used to compare exercised and sedentary P301S mice. A two-way ANOVA was used to compare all four groups (Tg-EX, Tg-SED, NTg-EX, and NTg-SED). After the ANOVA, a Newman-Keuls *post-hoc* (for behavioral tests; [125]) or Tukey’s HSD *post-hoc* was used to compare the significant effects between groups. All results are displayed as mean ± SEM.

## Abbreviations

AD: Alzheimer’s disease
CNS: Central nervous system
NFT: Neurofibrillary tangle
Aβ: Amyloid beta
NTg: Non-transgenic
Tg: Transgenic
EX: Exercised
SED: Sedentary
LC3: Microtubule-associated protein 1A/1B light chain 3
p62/SQSTM1: p62/sequestosome 1
CA3: Cornus ammonis 3
CA1: Cornus ammonis 1.
